# Toxicity of Aqueous L-Selenomethionine and Tert-Butyl Hydroperoxide Exposure to Zebrafish (*Danio rerio*) Embryos Following Tert-Butyl Hydroquinone Treatment

**DOI:** 10.3390/toxics7030044

**Published:** 2019-08-29

**Authors:** Allyson K. Gerhart, David M. Janz

**Affiliations:** 1 Toxicology Graduate Program, University of Saskatchewan, Saskatoon, SK S7N 5B3, Canada; 2 Department of Veterinary Biomedical Sciences, University of Saskatchewan, Saskatoon, SK S7N 5B4, Canada

**Keywords:** zebrafish, embryo, selenomethionine, tert-butyl hydroperoxide, tert-butyl hydroquinone, aqueous, deformities

## Abstract

Aqueous L-selenomethionine (SeMet) embryo exposures represent a rapid and simplified method for investigating the embryotoxic effects of SeMet. Using zebrafish (*Danio rerio*) as a model organism, the objective of the present study was to characterize the effects of waterborne exposure to both SeMet and tert-butyl hydroperoxide (tBOOH) to early life stages of zebrafish pre-treated with the antioxidant tert-butyl hydroquinone (tBHQ) in an attempt to investigate the mechanism of Se toxicity as it relates to oxidative stress. During the initial concentration range finding experiment, recently fertilized embryos were exposed for five days to 5, 25, 125, and 625 µg Se/L (as SeMet). These exposures informed the second experiment in which embryos were exposed to two concentrations of SeMet (25 and 125 µg Se/L) and 75 mg/L tBOOH either with (tBOOH-t, 25-t, 125-t) or without (tBOOH, 25, 125) a 4 h 100 µg/L tBHQ pre-treatment. Survival, hatchability, time to hatch, the frequency and severity of deformities (total and type), and changes in the expression of seven antioxidant-associated genes were determined. Exposures to SeMet and tBOOH reduced hatchability, increased time to hatch, decreased survival, increased the incidence and severity of deformities, and increased glutathione-disulfide reductase (*gsr*) expression in the pre-treated tBOOH treatment group.

## 1. Introduction

The zebrafish (*Danio rerio*) represents one of the most widely-used small fish models in toxicological research, due to beneficial traits such as a short life cycle, frequent egg production, ease of culture, and a transparent embryo chorion which allows for the determination of fertilization status and morphological analysis throughout development [1]. However, the transparent chorion, along with the highly detailed accounts of embryonic development and the substantial volume of toxicity data available in the literature for this species, makes zebrafish embryos an ideal candidate for studying the effects of contaminants with known developmental effects such as selenium (Se).

In most vertebrate organisms, Se is an essential micronutrient due to its role in the synthesis of proteins and enzymes involved in redox homeostasis [2]. However, the narrow range between essentiality and toxicity of Se is particularly concerning to oviparous vertebrates, which exhibit an increased susceptibility to Se during early life stages [3]. Exposure to elevated concentrations of selenomethionine (SeMet), the predominant form of Se in the diet, during the sensitive, early life stages often results in an increased incidence of edema and mortality, as well as teratogenic effects such as spinal/skeletal deformities, misshapen or missing fins, and craniofacial malformations in developing fish larvae [3,4]. These physical abnormalities are ecotoxicologically relevant as they can directly impair the fishes’ ability to swim, feed, and reproduce, which will ultimately lead to diminished population size and diversity over time [5].

While the relationship between Se exposure and the production of deformities is well established, the mechanism behind the manifestation of these physical abnormalities is still unclear and lacks consensus. Two main theories exist regarding the mechanism of Se toxicity, including Se substitution for sulfur in sulfur-containing amino acids/biomolecules, leading to improper protein folding and function, and the overproduction of reactive molecules as a result of SeMet metabolism, leading to an overwhelmed antioxidant defense system and consequently oxidative stress [6]. However, oxidative stress seems to have received the most attention in the literature [7,8,9].

Previous research investigating the embryotoxic effects of Se mainly involved dietary exposures [6], yolk microinjections [10], and more recently, aqueous embryo exposures [11,12,13,14,15]. A previous aqueous SeMet embryo exposure study in zebrafish focused on clarifying the relationship between oxidative stress and Se toxicity employed an antioxidant rescue methodology in which a subset of embryos were pre-treated for 24 h with the antioxidant N-acetylcysteine (NAC) prior to SeMet exposure [12]. Based on this concept, as well as previous research using electrophilic compounds that elicit an oxidative stress response similar to SeMet (i.e., tert-butyl hydroperoxide (tBOOH) and tert-butyl hydroquinone (tBHQ)) [16,17], we attempted to design and conduct aqueous embryo exposures that would further clarify the relationship between oxidative stress and the manifestation of deformities in larval fish. Therefore, tBOOH, which is an organic peroxide, was selected as a positive control as it should induce oxidative stress [17]. Similarly, tBHQ, which is a synthetic, organic antioxidant, was selected for use as an antioxidant [16]. Both chemicals were used previously in research focused on understanding the antioxidant defense system in mammalian cells, as they were found to trigger an oxidative stress response through activation of Nrf2, which consequently activates the transcription of phase II and antioxidant enzymes [16,17]. Therefore, we predicted that pre-treating with tBHQ would activate/prepare the antioxidant defense system prior to exposure to SeMet or tBOOH, and thus serve a protective function.

As such, the objective of this research was to further investigate the mechanism of Se toxicity, as it relates to oxidative stress, by characterizing the effects of waterborne SeMet, tBOOH, and tBHQ, alone and in combination, on the hatchability, mortality, incidence/severity of deformities, and expression of seven genes relevant to the antioxidant defense system in developing zebrafish embryos.

## 2. Materials and Methods

### 2.1. Chemicals

Seleno-L-methionine (SeMet; ≥98% purity), tert-Butyl hydroquinone (tBHQ; 97% purity), and tert-Butyl hydroperoxide (tBOOH; 70% solution in water) were purchased from Sigma-Aldrich (Oakville, ON, Canada).

### 2.2. Zebrafish Embryos

Newly fertilized zebrafish embryos were collected in house from an adult breeding colony maintained in an environmental chamber with controlled temperature (27 +/− 1 °C) and photoperiod (16:8 light:dark) at the Toxicology Centre, University of Saskatchewan (Saskatoon, SA, Canada). Embryos determined to be healthy/developmentally normal and within the 4-cell–high blastula stage (i.e., within 3 h of fertilization) of embryonic development [18] were selected for use in experiments. Animal care and all experimentation were conducted in compliance with the University Committee on Animal Care and Supply (UCACS) and was approved by the Animal Research Ethics Board (AREB) (Animal Use Protocol #20030076, approved May 30, 2018).

### 2.3. Aqueous Selenomethionine Dose-Response Embryo Exposures

Embryos were randomly distributed into glass petri dishes containing 40 mL of test solution. Each petri dish contained 20 embryos, with 9–12 replicate petri dishes in each exposure group. Exposure proceeded for 5 days in facility water alone (control embryos) or facility water spiked with nominal SeMet concentrations of 5, 25, 125, and 625 µg Se/L. To maintain water quality, 75% solution changes were performed daily and any dead embryos were removed.

### 2.4. Aqueous Selenomethionine, Pro- and Antioxidant Embryo Exposures

The 100 µg/L concentration of tBHQ was chosen based on preliminary studies as a concentration that did not induce a rate of mortality or deformities greater than that of the control embryos (unpublished data), while 75 mg/L of tBOOH was chosen based on preliminary studies as a concentration that caused a significant increase in the rate of deformities (unpublished data) relative to control embryos. Based on the aqueous SeMet exposures, 25 and 125 µg Se/L were selected for use in the following exposures as concentrations that caused a significant elevation in both mortality and the frequency of deformities relative to control embryos.

Zebrafish embryos were collected and sorted as described previously. Embryos receiving the antioxidant pre-treatment were randomly distributed into plastic petri dishes containing 40 mL of 100 µg/L tBHQ, while embryos not receiving the pre-treatment were randomly distributed into plastic petri dishes containing 40 mL of facility water. Following 4 h, all embryos were re-distributed into glass petri dishes containing 40 mL of facility water alone, 75 mg/L tBOOH, 25 µg Se/L, or 125 µg Se/L. This resulted in 8 treatment groups, including: facility water alone (control embryos), 100 µg/L tBHQ (tBHQ control), 75 mg/L of tBOOH with and without antioxidant pre-treatment (denoted as tBOOH-t and tBOOH, respectively), 25 µg Se/L with and without antioxidant pre-treatment (denoted as 25-t and 25, respectively), and 125 µg Se/L with and without antioxidant pre-treatment (denoted as 125-t and 125, respectively). Each individual petri dish contained 20 embryos, with 32–33 control replicates and 13–21 replicates in each exposure group. Exposure proceeded for 5 days, during which dead embryos were removed, and 75% solution changes were performed daily.

Following the 5-day aqueous exposures, remaining live larvae destined for deformities analysis were euthanized with an overdose of buffered MS-222 (250 mg/L, pH 7.0–7.4), fixed in buffered 10% formalin for 18–24 h, and stored in 70% ethanol. The preserved larvae were then examined for malformations in a blind fashion using an Olympus model S261 dissecting microscope (Olympus, Melville, NY, USA). Larvae destined for biochemical analysis were frozen at −80 °C until analysis.

### 2.5. Deformities Analysis

In the present study, both the frequency and severity of larval deformities were quantified. During frequency analysis, larvae in each replicate were individually assessed and categorized as either deformed or not deformed. An overall incidence was calculated by dividing the number of individuals classified as deformed by the total number of individuals in that replicate. This process was then repeated for specific types of deformities (kyphosis, scoliosis, craniofacial, finfold, and edema).

The severity of abnormalities was assessed using the Graduated Severity Index (GSI) system [15,19]. Severity scores ranged from 0–2, where scores of 0 = normal, 1 = moderate, and 2 = severe. Total GSI (overall severity of deformities in each treatment) was calculated by summing the severity score assigned to each category (kyphosis, scoliosis, craniofacial, finfold, and edema) for each individual fry in the replicate. The sums calculated for each fry were then averaged to obtain an overall severity for each replicate. In addition to total GSI, the severity of each distinct type of deformity was also calculated. This was calculated by taking the average of the severity scores assigned to each fry in the category of interest (either kyphosis, scoliosis, craniofacial, finfold, or edema) in each replicate.

### 2.6. Water and Tissue Concentrations

Water samples taken from the exposure stock solutions were filtered and acidified with 2% high purity nitric acid (Fisher Scientific, Hampton, NH, USA) for total Se analysis. Pooled larvae collected for total Se analysis were euthanized with MS-222, collected in microcentrifuge tubes and stored at −20 °C until they could be freeze dried. Dried samples were then digested using high purity nitric acid and 30% hydrogen peroxide. Inductively coupled plasma-mass spectrometry (ICP-MS) (8800 ICP-MS Triple Quad, Agilent Technologies, Santa Clara, CA, USA) was used to quantify total Se in both water and tissue samples as described previously [10]. Instrument performance was verified using the standard reference material 1640a solution (National Institute of Standards and Technology). Analysis of the SeMet stock solutions used in the aqueous embryo exposures produced an average percent recovery of 102% (SD; ±4.2%). Analysis of tissue samples yielded a 1640a recovery of 102% (SD; ±1.3%). The tissue digestion process/method was verified using a certified reference material (TORT-2, lobster hepatopancreas, NRC, Ottawa, ON, Canada) for which a recovery of 103% (SD; ±8.1%) was obtained. Instrument detection limit differed in each run, with detection limits ranging from 0.006–0.102 µg/L for water samples and 0.061 mg/kg for tissue samples.

### 2.7. Real-Time Quantitative PCR (RT-qPCR)

Four to five replicates of 11–20 zebrafish larvae exposed to the SeMet, pro- and antioxidant treatment combinations described previously were collected in microcentrifuge tubes and stored at −80 °C until analysis. Total RNA was extracted using the RNeasy Plus Universal Mini Kit (QIAGEN Inc., Toronto, ON, Canada) according to the manufacturer’s protocol, quantified using the QIAxpert® microfluidic UV/VIS Spectrophotometer (QIAGEN Inc., Toronto, ON, Canada), and stored at −80 °C until analyzed. First-strand cDNA synthesis was performed using the QuantiNova™ Reverse Transcription Kit (QIAGEN) according to the manufacturer’s protocol using 2.25 µg of total RNA. The cDNA samples were stored at −20 °C until analyzed. Quantitative Real-Time PCR was performed in optical, fast, clear 96-well plates using the PowerUp™ SYBR™ Green Master Mix qPCR Kit (Applied Biosystems, Foster City, CA, USA) and the QuantStudio™ 6 Flex Real-Time PCR System (Applied Biosystems).

A 25 µL reaction mixture containing PowerUp™ SYBR™ Green Master Mix (Applied Biosystems), an optimized concentration of cDNA (100 ng) and gene-specific qPCR primers (0.6 µmol), as well as nuclease free water was used for each sample and primer combination. The PCR reaction mixture was incubated/denatured at 50 °C for 2 min, then 95 °C for 2 min before the first PCR cycle. The thermal cycle profile consisted of denaturing at 95 °C for 1 s and extension at 60 °C for 30 s for a total of 40 PCR cycles. This was immediately followed by a dissociation curve. Each reaction was conducted in duplicate. Target gene primer sequences, accession numbers, and efficiencies for nuclear factor erythroid 2-related factor 2 (*nrf2a*), glutathione peroxidase 1a (*gpx1a*), glutathione s-transferase piscine 1 (*gst p1*), glutathione-disulfide reductase (*gsr*), manganese superoxide dismutase (*sod2*), glutamate-cysteine ligase catalytic subunit (*gclc*), aryl hydrocarbon receptor 2 (*ahr2*), actin beta (*actb*), and elongation factor 1 alpha (*ef1α*) are shown in Table S1 [20,21]. Quantitative real-time PCR data was analyzed and quantified using the QuantStudio™ Real-Time PCR Software v1.3 (Applied Biosystems). Relative changes in target gene expression was determined using the 2^−∆∆CT^ method described by Applied Biosystems [22], with *actb* and *ef1α* serving as reference genes. A slight deviation from the suggested calculations involved the use of the control group’s mean ΔC_T_ to calculate a ΔΔC_T_ value and fold change value for each replicate as opposed to each treatment group, allowing for the calculation of error terms.

### 2.8. Statistics

All results were reported as mean ± standard error, and all analyses were performed using GraphPad Prism Version 8.1.2 (GraphPad Software: La Jolla, CA, USA). All data were tested for normality and homogeneity of variance using the Shapiro-Wilk normality test and either the Browne-Forsythe test or Bartlett’s test as appropriate. When data distribution was normal and exhibited homogeneity of variance, one-way analysis of variance (ANOVA) followed by Holm-Sidak’s multiple comparisons test was used to test for significant differences between treatment groups. When data distribution was non-normal, or data could not be satisfactorily transformed, a non-parametric Kruskal–Wallis one-way ANOVA by ranks was used followed by Dunn’s multiple comparisons test. Alpha values were two-tailed and set at 0.05. A nonlinear regression, dose-response analysis without constraints was performed on the percent cumulative mortality and deformities data to determine an LC_50_ and EC_50_ following SeMet exposure.

## 3. Results

### 3.1. Aqueous Selenomethionine Dose-Response Embryo Exposures

#### 3.1.1. Tissue Concentrations

Total Se quantified in pooled larvae following 5 days of aqueous exposure at concentrations of 5, 25, 125, and 625 µg Se/L had mean total Se tissue concentrations of 34.2, 144, 264, and 291 µg Se/g dry mass (d.m.), respectively, compared to 2.98 µg/g d.m. in control embryos, indicating bioaccumulation in larvae in a concentration-dependent manner. Test solutions were also analyzed for total Se using ICP-MS to verify exposure concentrations (Table 1). Measured concentrations were all in agreement with nominal concentrations.

#### 3.1.2. Mortality

**Figure 1:**
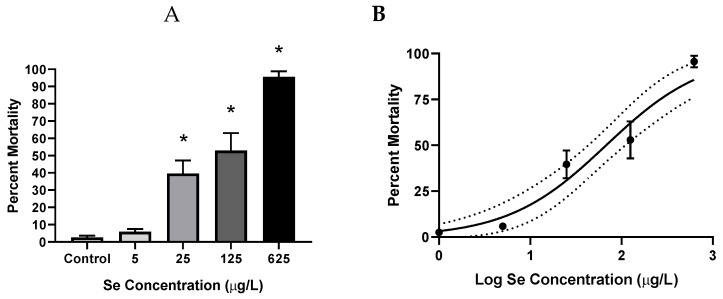
Mean (±SE) percent cumulative mortality (1–5 dpf) of zebrafish exposed to increasing concentrations of SeMet via embryo aqueous exposure. (**A**) Bar graph, asterisks represent significant differences compared to the control using a Kruskal–Wallis one-way analysis of variance (ANOVA) by ranks followed by a Dunn’s multiple comparisons test (*p* < 0.05); *n* = 8–12 replicates of 20 embryos. (**B**) Dose-response curve used to calculate the LC_50_ value of 67.9 µg/L (95% CI = 43.4–108 µg/L). Dotted lines represent the 95% confidence interval.

SeMet exposures ≥ 25 µg/L significantly decreased embryo survival, with percentages of 39.6% ± 7.6%, 52.9% ± 10.1%, and 95.6% ± 3.4% (*p* ≤ 0.024), compared to 2.50% ± 1.2% in the controls. An LC_50_ value of 67.9 µg/L (95% CI = 43.4–108 µg/L) was calculated for SeMet-induced mortality (Figure 1). From a time-course perspective, embryo mortality started to increase on the third day, reached a maximum on the 4th day, and was usually followed by a slight decrease on the 5th day of exposure.

#### 3.1.3. Frequency of Deformities

**Figure 2:**
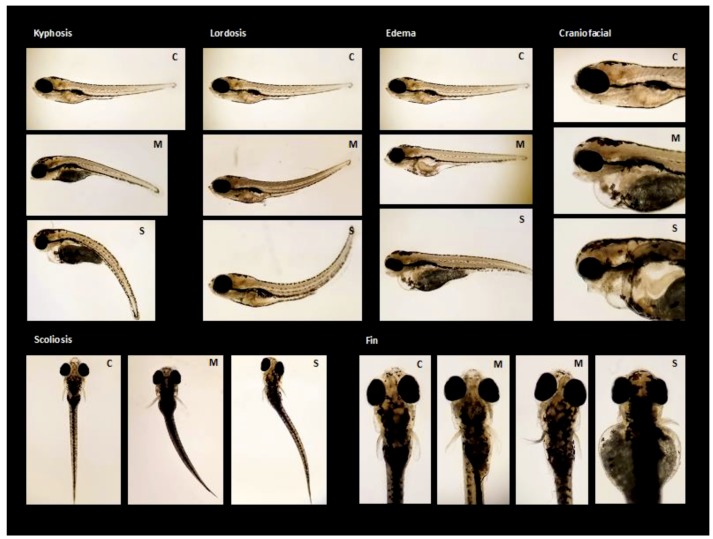
Morphological malformations at varying degrees of severity observed in larval zebrafish exposed to increasing concentrations of L-selenomethionine (SeMet) via embryo aqueous exposure during deformities analysis. Letters indicate level of severity; Control (C), Moderate (M), and Severe (S).

**Figure 3:**
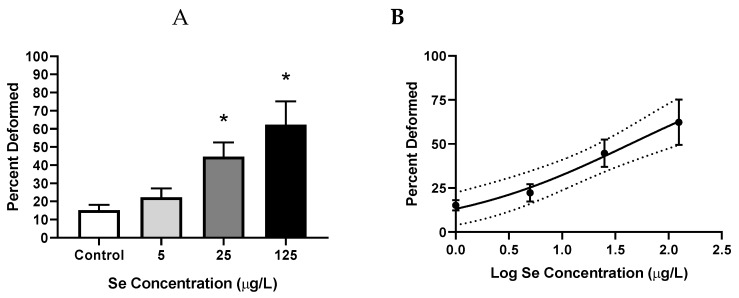
Mean (±SE) percentage of total deformities (sum of skeletal, craniofacial, finfold, and edema) in larval zebrafish exposed to increasing concentrations of L-selenomethionine (SeMet) via embryo aqueous exposure. (**A**) Bar graph, asterisks represent significant differences from control using a Kruskal–Wallis one-way analysis of variance (ANOVA) by ranks followed by a Dunn’s multiple comparisons test (*p* < 0.05); *n* = 9–12 replicates of 20 embryos. (**B**) Dose-response curve used to calculate the EC_50_ value of 43.8 µg/L (95% CI = 21.4–138 µg/L). Dotted lines represent the 95% confidence interval.

Representative images of the deformities observed in this study can be found in Figure 2. SeMet exposures ≥25 µg/L significantly increased the percentage of total deformities, with percentages of 44.7% ± 7.8% (*p* = 0.019) and 62.3% ± 14% (*p* ≤ 0.0185) compared to 15.2% ± 2.9% in the controls. An EC_50_ value of 43.8 µg/L (95% CI = 21.4–138 µg/L) was also calculated for SeMet-induced deformities (Figure 3).

In addition to total deformities, the incidence of edema, craniofacial and fin malformations, as well as spinal curvatures (kyphosis and scoliosis) were also calculated. While exposure to SeMet had no significant effect on the incidence of skeletal deformities in zebrafish larvae, the incidence of edema, fin, and craniofacial malformations increased in a concentration-dependent manner, with significant elevations observed at concentrations ≥25 µg/L (*p* ≤ 0.0098). The incidence of edema, craniofacial and fin malformations in the 25 and 125 µg/L treatments were 25.9% ± 7.2% and 39.4% ± 9.4% compared to 0.417% ± 0.42% in the controls; 28.3% ± 8.2% and 30.7% ± 7.2% compared to 3.38% ± 1.4% in the controls; and 31.9% ± 6.3% and 55.1% ± 16% compared to 7.15% ± 2.2% in the controls, respectively (Figure S1).

#### 3.1.4. Severity of Deformities

**Figure 4:**
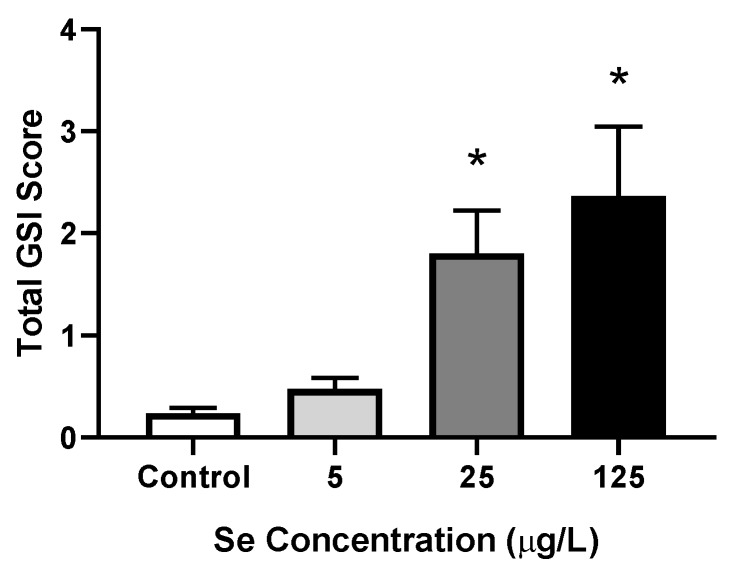
Mean (±SE) graduated severity index (GSI) scores for total deformities (sum of skeletal, craniofacial, finfold, and edema scores) in larval zebrafish exposed to increasing concentrations of L-selenomethionine (SeMet) via embryo aqueous exposure. Asterisks represent significant differences compared to the control using a one-way ANOVA followed by a Dunnett’s multiple comparisons test (*p* < 0.05); *n* = 9–12 replicates of 20 embryos.

In addition to frequency, the severity of deformities was also evaluated. At concentrations ≥25 µg/L a significant increase in the severity of deformities was observed, with total GSI scores of 1.80 ± 0.42 and 2.36 ± 0.72 in 25 and 125 µg/L, respectively (*p* ≤ 0.0036), compared to 0.237 ± 0.056 in the control larvae (Figure 4).

The severity of individual deformity categories was also assessed. While the severity of skeletal deformities did not significantly differ following exposure, the severity of edema, fin, and craniofacial malformations increased in a concentration-dependent manner, with significant elevations observed at concentrations ≥25 µg/L (*p* ≤ 0.0095). The severity/GSI scores for edema, craniofacial and fin malformations in the 25 and 125 µg/L treatments were: 0.383 ± 0.11 and 0.460 ± 0.11 compared to 0.004 ± 0.004 in the controls; 0.332 ± 0.082 and 0.307 ± 0.072 compared to 0.0338 ± 0.014 in the controls; and 0.472 ± 0.10 and 0.743 ± 0.23 compared to 0.0882 ± 0.030 in the controls, respectively (Figure S2).

#### 3.1.5. Hatchability and Time to Hatch

SeMet exposure had no significant effect on mean time to hatch (Figure S3); however, a significant reduction in embryo hatchability was observed at concentrations ≥25 µg/L, with percentages of 61.3% ± 8.7%, 60.0% ± 8.1%, and 24.4% ± 8.0% (*p* ≤ 0.0073) compared to 97.5% ± 1.2% in the controls (Figure S4).

### 3.2. Aqueous Selenomethionine, Pro- and Antioxidant Embryo Exposures

#### 3.2.1. Mortality 

**Figure 5:**
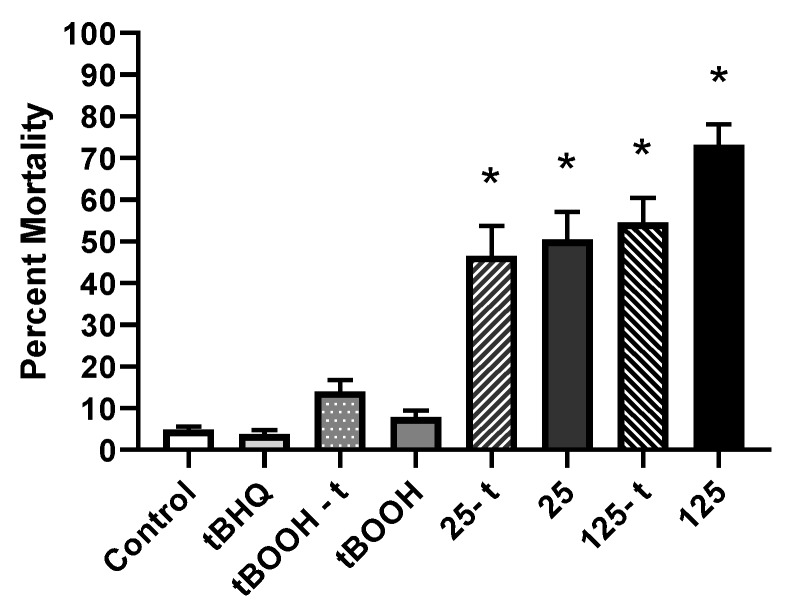
Mean (±SE) percent cumulative mortality (1–5 dpf) of zebrafish exposed via embryo aqueous exposure to facility water (control), tBHQ, tBOOH, and two concentrations of SeMet (25 and 125 µg Se/L) either with (tBOOH-t, 25-t, 125-t) or without (tBOOH, 25, 125) a tBHQ pre-treatment. Asterisks represent significant differences compared to the control using a Kruskal–Wallis one-way analysis of variance (ANOVA) by ranks followed by a Dunn’s multiple comparisons test (*p* < 0.05); *n* = 20–33 replicates of 20 embryos.

Embryos exposed to all SeMet treatments exhibited a significant increase in mortality compared to control embryos (*p* < 0.0001), with percentages of 46.5% ± 7.2%, 50.5% ± 6.6%, 54.5% ± 5.9%, and 73.1% ± 5.0% in the 25-t, 25, 125-t, and 125 treatments, respectively, compared to 4.85% ± 0.74% in the controls. However, no differences were observed between embryos pre-treated with tBHQ and embryos exposed to SeMet alone (Figure 5).

#### 3.2.2. Frequency of Deformities

**Figure 6:**
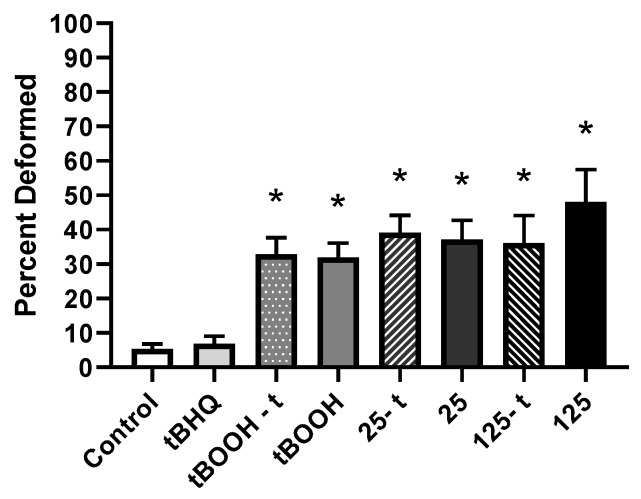
Mean (±SE) percentage of total deformities (sum of skeletal, craniofacial, finfold, and edema) in larval zebrafish exposed via embryo aqueous exposure to facility water (control), tBHQ, tBOOH, and two concentrations of SeMet (25 and 125 µg Se/L) either with (tBOOH-t, 25-t, 125-t) or without (tBOOH, 25, 125) a tBHQ pre-treatment. Asterisks represent significant differences from control using a Kruskal–Wallis one-way analysis of variance (ANOVA) by ranks followed by a Dunn’s multiple comparisons test (*p* < 0.05); *n* = 13–33 replicates of 20 embryos.

A significant increase in the proportion of deformities was observed in all treatment groups (*p* < 0.0012), with percentages of 6.86% ± 2.2%, 32.9% ± 4.9%, 31.9% ± 4.3%, 39.1% ± 5.1%, 37.1% ± 5.6%, 36.2% ± 8.0%, and 48.1% ± 9.4% in the tBOOH-t, tBOOH, 25-t, 25, 125-t, and 125 treatments respectively, compared to 5.30% ± 1.5% in the controls. No significant differences were observed between embryos pre-treated with tBHQ and embryos exposed to either tBOOH or SeMet alone (Figure 6).

The incidence of kyphosis increased significantly within all SeMet treatment groups (*p* ≤ 0.045), with percentages of 7.36% ± 2.9%, 8.62% ± 3.3%, 12.8% ± 5.2%, and 25.6% ± 9.3% in the 25-t, 25, 125-t, and 125 treatments, respectively, compared to 0.479% ± 0.27% in the controls. Scoliosis only showed a significant increase in frequency in the pre-treated 25 µg/L group (*p* = 0.0072), with a percentage of 15.1% ± 3.5% compared to 3.71% ± 1.4% in the controls. The incidence of edema increased significantly in all treatment groups (*p* ≤ 0.025), with percentages of 15.5% ± 4.6%, 21.3% ± 4.4%, 26.3% ± 5.2%, 34.7% ± 5.6%, 24.0% ± 6.2%, and 30.6% ± 8.4% in the tBOOH-t, tBOOH, 25-t, 25, 125-t, and 125 treatments, respectively, compared to 0.470% ± 0.26% in the controls. The incidence of fin malformations increased significantly in the pre-treated tBOOH group and within all SeMet treatment groups compared to the controls (*p* ≤ 0.017), with percentages of 14.8% ± 3.1%, 17.9% ± 5.7%, 17.0% ± 3.6%, 14.4% ± 4.1%, and 27.0% ± 8.0% in the tBOOH-t, 25-t, 25, 125-t, and 125 treatments, respectively, compared to 1.58% ± 0.62% in the controls. Finally, craniofacial malformations increased significantly in frequency in both 25 µg/L SeMet treatment groups (*p* ≤ 0.022), with percentages of 11.2% ± 3.9% and 11.0% ± 3.2% in the 25-t and 25 treatments, respectively, compared to 0.614% ± 0.36% in the controls. No significant differences were observed between embryos pre-treated with tBHQ and embryos exposed to either tBOOH or SeMet alone (Figure S5).

#### 3.2.3. Severity of Deformities

**Figure 7:**
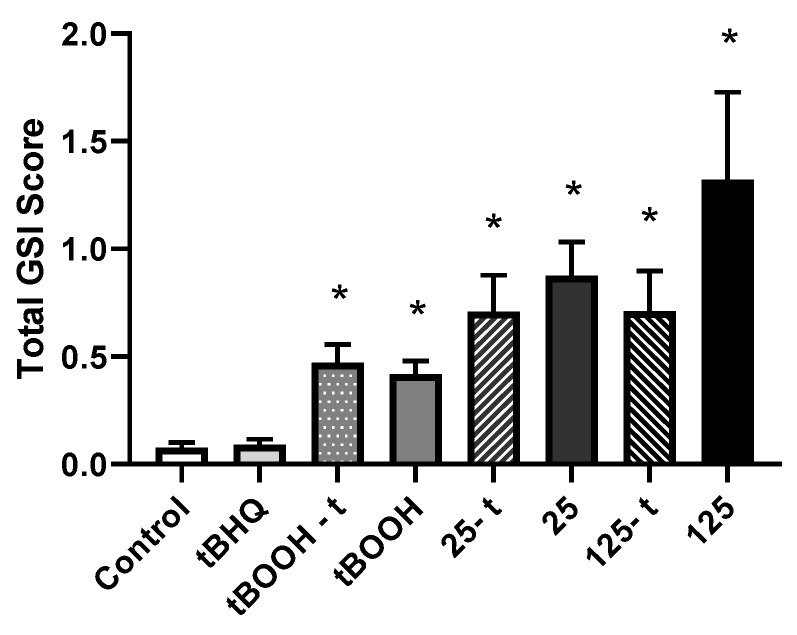
Mean (±SE) graduated severity index (GSI) scores for total deformities (sum of skeletal, craniofacial, finfold, and edema scores) in larval zebrafish exposed via embryo aqueous exposure to facility water (control), tBHQ, tBOOH, and two concentrations of SeMet (25 and 125 µg Se/L) either with (tBOOH-t, 25-t, 125-t) or without (tBOOH, 25, 125) a tBHQ pre-treatment. Asterisks represent significant differences from control using a Kruskal–Wallis one-way analysis of variance (ANOVA) by ranks followed by a Dunn’s multiple comparisons test (*p* < 0.05); *n* = 13–33 replicates of 20 embryos.

Exposure to all treatment groups resulted in a significant increase in the severity of deformities as compared to control larvae (*p* ≤ 0.0010), with severity/GSI scores of 0.470 ± 0.086, 0.417 ± 0.062, 0.709 ± 0.17, 0.875 ± 0.16, 0.710 ± 0.19, and 1.32 ± 0.41 in the tBOOH-t, tBOOH, 25-t, 25, 125-t, and 125 treatments, respectively, compared to 0.0766 ± 0.024 in the controls. However, no significant differences were observed between embryos pre-treated with tBHQ and embryos exposed to either tBOOH or SeMet alone (Figure 7).

The severity of kyphosis increased significantly in the 25 µg/L group as well as both 125 µg/L treatment groups (*p* ≤ 0.037), with severity/GSI scores of 0.0991 ± 0.035, 0.142 ± 0.053, and 0.340 ± 0.13 in the 25, 125-t, and 125 treatments, respectively, compared to 0.0048 ± 0.003 in the controls. The severity of scoliosis only increased significantly in the pre-treated 25 µg/L group (*p* ≤ 0.020), with a severity/GSI score of 0.162 ± 0.038 compared to 0.0422 ± 0.015 in the controls. Exposure to all treatment groups resulted in a significant increase in the severity of edema (*p* ≤ 0.030), with severity/GSI scores of 0.155 ± 0.046, 0.213 ± 0.044, 0.229 ± 0.044, 0.375 ± 0.061, 0.240 ± 0.062, and 0.337 ± 0.092 in the tBOOH-t, tBOOH, 25-t, 25, 125-t, and 125 treatments, respectively, compared to 0.0062 ± 0.004 in the controls. In contrast, the severity of craniofacial malformations increased significantly only in the 25 µg/L group (*p* ≤ 0.0080), with severity/GSI score of 0.110 ± 0.032 compared to 0.0077 ± 0.005 in the controls. Finally, the severity of fin malformations increased significantly within the pre-treated tBOOH group, 25 µg/L group, and both 125 µg/L treatment groups (*p* ≤ 0.014), with severity/GSI scores of 0.151 ± 0.031, 0.205 ± 0.052, 0.178 ± 0.063, and 0.351 ± 0.12 in the tBOOH-t, 25, 125-t, and 125 treatments, respectively, compared to 0.0158 ± 0.0062 in the controls. No significant differences were observed between embryos pre-treated with tBHQ and embryos exposed to either tBOOH or SeMet alone (Figure S6).

#### 3.2.4. Hatchability and Time to Hatch 

Significant increases in the mean time to hatch were observed between control embryos and those in both tBOOH treatment groups, as well as the pre-treated 25 µg/L group and 125 µg/L group (*p* ≤ 0.045), with mean time to hatch values of 3.90 ± 0.13 days, 3.79 ± 0.11 days, 3.66 ± 0.11 days, and 3.58 ± 0.083 days in the tBOOH-t, tBOOH, 25-t, and 125 treatments, respectively, compared to 3.29 ± 0.045 days in the controls (Figure S7). Furthermore, exposure to all treatment groups resulted in a significant reduction in hatchability relative to control embryos (*p* ≤ 0.078), with percentages of 40.7% ± 5.8%, 70.7% ± 5.5%, 51.0% ± 7.3%, 68.1% ± 5.2%, 47.0% ± 6.5%, and 38.6% ± 4.4% in the tBOOH-t, tBOOH, 25-t, 25, 125-t, and 125 treatments, respectively, compared to 95.2% ± 0.74% in the controls. No significant differences in time to hatch or hatchability were observed between embryos pre-treated with tBHQ and embryos exposed to either tBOOH or SeMet alone (Figure S8).

#### 3.2.5. Gene Expression

Aside from a significant 6.96-fold increase in *gsr* expression relative to control embryos in the pre-treated tBOOH treatment group, exposure did not significantly affect the expression of the other genes selected. Although not statistically significant, *gsr* also appeared to show a trend towards increased expression following exposure to the 125-t and 125 µg/L treatment groups, with 3.99-fold and 4.23-fold increases in expression relative to control embryos, respectively (*p* = 0.104 for both). Consistent with all previous endpoints, no significant differences were observed between embryos pre-treated with tBHQ and embryos exposed to either tBOOH or SeMet alone (Figure S9).

## 4. Discussion

### 4.1. Aqueous Selenomethionine Dose-Response Embryo Exposures

#### 4.1.1. Total Selenium in Stock Solutions and Tissue

As was mentioned previously, earlier research addressing the embryotoxic effects of Se chiefly consisted of maternal dietary exposures [6] and yolk microinjections [10]. Despite temporal and species-specific variation in chorion permeability to waterborne contaminants, aqueous embryo exposures [11,12,13,14,15] have begun to gain traction as they offer a more simplified and rapid method for evaluating the effects of SeMet on developing fish embryos. Previous aqueous embryo exposure research suggests that the chorion does exhibit some degree of permeability to SeMet, as embryos displayed increased Se tissue concentrations following aqueous exposure [14,15]. This was consistent with findings in the present study.

#### 4.1.2. Mortality

Previous aqueous SeMet embryo exposure work in zebrafish [12], Japanese medaka (*Oryzias latipes*) [13], and fathead minnow (*Pimephales promelas*) [15] reported significant elevations in mortality at 100, 490, and 810 µg SeMet/L, respectively. Thus, based on the present study and a previous report [12], it appears zebrafish embryos are more sensitive to aqueous SeMet exposures than medaka and fathead minnow.

#### 4.1.3. Frequency and Severity of Deformities

The heightened sensitivity of zebrafish relative to other fish species used in aqueous embryo SeMet exposure experiments remained consistent during evaluations of larval deformities, as previous aqueous SeMet embryo exposure work in Japanese medaka [13] and fathead minnow [15] only reported significant elevations in the frequency of deformities at concentrations of 490 and 810 µg SeMet/L, respectively. Further analysis of the incidence of specific categories of deformities suggested that exposure to SeMet had no significant effect on the incidence of skeletal deformities, despite a concentration-dependent increase in the incidence of edema, fin, and craniofacial malformations. While this was consistent with previous work in fathead minnows [15], previous studies suggested that spinal deformities are the most representative and common type of deformity observed in Se-exposed embryos [14,23].

Consistent with the mortality and deformities frequency data, comparisons between the present study and previous aqueous embryo exposure work suggest that zebrafish are more sensitive to aqueous Se exposures, as our previous work in fathead minnows [15] showed a significant increase in the severity of deformities starting at 810 µg/L compared to 25 µg/L in the present study. Furthermore, the increased responsiveness of edema and fin/craniofacial malformations (to SeMet exposure) relative to spinal deformities, as illustrated by severity, was also observed in fathead minnows [15]. Further investigation into the effect of differing methods of aqueously exposing embryos (i.e., timing, concentrations, etc.) would be required to determine why these types of deformities were not only more common, as observed during frequency analysis, but also more severe in both our fathead minnow and zebrafish embryos compared to previous aqueous embryo exposure studies.

#### 4.1.4. Hatchability and Time to Hatch

The lack of effect on time to hatch is consistent with previous work in fathead minnow [15] and Japanese medaka [14] embryos, where significant reductions in mean/median time to hatch was only observed at 65610 µg/L (the highest exposure concentration) and 5 µM SeMet (1000 µg/L) at stage 34, respectively. Effects on hatchability were also consistent with the aqueous Se embryo exposure literature. In fathead minnows exposed to Se (as SeMet) concentrations between 30 and 65610 µg/L, embryo hatchability was significantly reduced at 21870 µg/L and 65610 µg/L [15]. Furthermore, Japanese medaka embryos aqueously exposed to Se (as SeMet) at concentrations of 0.5, 5, and 50 µM (approximately 100, 1000, and 10000 µg/L) at six developmental stages reported significantly reduced hatchability at concentrations ≥5 µM SeMet at all stages of development [15]. Another study involving Japanese medaka exposed aqueously to 0.05 mM Se (as SeMet) (approximately 10000 µg Se/L) in freshwater and three different hypersaline conditions also reported significant reductions in hatchability in all treatment groups [11].

### 4.2. Aqueous Selenomethionine, Pro- and Antioxidant Embryo Exposures

Based on the results of the first experiment, 25 and 125 µg Se/L were selected for use in the second experiment, along with two additional pharmacological agents, tert-butyl hydroperoxide (tBOOH), and tert-butyl hydroquinone (tBHQ).

#### 4.2.1. Mortality

Consistent with the first experiment, exposure to the 25 and 125 µg/L SeMet treatment concentrations caused a significant increase in mortality compared to control embryos; however, no differences were observed between embryos pre-treated with tBHQ and embryos exposed to SeMet alone. This was consistent with previous aqueous SeMet embryo exposure work in zebrafish, where embryos were pre-treated with the antioxidant NAC for 24 h prior to SeMet exposure and no significant differences in mortality compared to embryos exposed to SeMet alone were observed [12]. As expected, exposure to the tBHQ did not result in significant mortality; however, the same was observed in both pro-oxidant tBOOH treatments. While this initially suggested the exposure concentration was too low, further evaluation of the deformities data suggested otherwise.

#### 4.2.2. Frequency and Severity of Deformities

Consistent with the first experiment, exposure to the 25 and 125 µg/L SeMet treatment concentrations also resulted in a significant increase in both the incidence and severity of deformities compared to control embryos. However, no differences were observed between embryos pre-treated with tBHQ and embryos exposed to SeMet alone. This contrasted with the antioxidant rescue work conducted by Arnold et al. [12], where embryos exposed to SeMet alone showed a significant increase in the frequency of deformities compared to controls and the embryos pre-treated with NAC for 24 h prior to SeMet exposure. Furthermore, embryos pre-treated with NAC showed a qualitative decrease in the severity of deformities [12]. As expected, exposure to tBHQ alone did not result in any significant increases in the incidence or severity of deformities. In terms of the tBOOH treatments, it is important to note that despite a lack of mortality following exposure, significant increases in both the incidence and severity of deformities were observed when compared to control embryos, as was in the SeMet treated embryos.

While there were no clear patterns in terms of the occurrence or severity of certain types of deformities observed, we did note that significant elevations in the incidence and severity of skeletal deformities was only observed in the SeMet treatments, and not the tBHQ controls or tBOOH treatments. Furthermore, there seemed to be a general trend towards a higher proportion and severity of deformities observed in the SeMet treatments compared with the tBOOH treatments.

In the present study, lordosis (a form of skeletal curvature) was excluded from the deformity calculations due to an abnormally high incidence within both the control and tBHQ treatment groups (Figure S10,11), as it was not believed to be reflective of Se exposure. Interestingly, the frequency of lordosis was lesser in the SeMet and tBOOH exposure groups compared to control and tBHQ groups. It is uncertain why the rate of lordosis was highest in both control treatments but seemed to decrease in frequency in the other treatment groups; however, it may have been related to either poor egg quality as a result of using an older zebrafish breeding stock, or some sort of nutritional deficiency.

#### 4.2.3. Hatchability and Time to Hatch

In the present study, no consistent patterns were evident during assessment of time to hatch. Furthermore, the biological relevance of these results was considered minimal, as the time to hatch in these treatments only differed from the controls by about a day at most. In terms of effects on hatchability, the reductions observed in the SeMet exposed embryos were consistent with the first experiment, as well as previous aqueous Se embryo work [11,14,15]. While exposure to tBHQ alone did not result in any significant changes in hatchability, reduced hatchability was observed in both tBOOH treatment groups. Of particular interest was the hatch rate in the pre-treated tBOOH group, as it was almost as severely reduced as embryos in the 125 µg/L Se treatment group (40.7% ± 5.8% compared to 38.6% ± 4.4%). Furthermore, it was noted during exposures that larvae in the tBOOH-t treatments generally appeared to be healthy and developmentally normal but appeared to struggle with hatching, inspiring more questions regarding the effect of waterborne exposures on physiochemical properties of the chorion.

#### 4.2.4. Gene Expression

In an attempt to further reinforce the relationship between oxidative stress and Se induced toxicity, changes in the expression of 7 genes relevant to the antioxidant defense system were evaluated. These included: nuclear factor erythroid 2-related factor 2 (*nrf2a*), which is an oxidant-responsive transcription factor that regulates the transcription of antioxidant genes such as gst p1 [17]; glutathione peroxidase 1a (*gpx1a*), which is an enzyme that serves a protective role in cells by catalyzing the GSH-dependent reduction/degradation of various hydroperoxides [24]; glutathione s-transferase piscine 1 (*gst p1*), which is one of the phase II detoxification enzymes that catalyze the conjugation of GSH to harmful/electrophilic compounds to aid with excretion [16,25]; glutathione-disulfide reductase (*gsr*), which is an enzyme that directly catalyzes the reduction of oxidized glutathione (GSSG) back to reduced glutathione (GSH) [26]; manganese superoxide dismutase (*sod2*), which is an enzyme that catalyzes the reduction of the superoxide anion radical (O_2_^∙-^) into hydrogen peroxide and oxygen [27]; glutamate-cysteine ligase catalytic subunit (*gclc*), which is a rate-limiting enzyme that catalyzes the first step in GSH synthesis from l-cysteine and glutamate; and finally aryl hydrocarbon receptor 2 (*ahr2*), which is a ligand-dependent transcription factor involved with the biotransformation of a range of contaminants [17,28].

As was mentioned previously, only *gsr* expression increased significantly out of the 7 genes evaluated, and only in the pre-treated tBOOH treatment group. Since neither tBOOH or tBHQ alone induced a response in *gsr*, it is difficult to conclude on which chemical was responsible for the up-regulation of this particular gene. Limited previous data exists for changes in *gsr* expression in fish embryos exposed to tBOOH or tBHQ; however, one study involving 4-dpf zebrafish eleutheroembryos (i.e., hatched but not yet free feeding embryos) exposed to tBHQ for 6 hr reported a significant increase in *gsr* gene expression [29].

## 5. Conclusions

Exposure to the tBOOH treatment groups did not cause significant mortality but did result in significant increases in both the incidence and severity of deformities such as those observed in the SeMet treated embryos, potentially suggesting a similar pathway (i.e., oxidative stress) is playing a role in the generation of deformities.

No differences were observed between embryos pre-treated with tBHQ and embryos exposed to either SeMet or tBOOH alone for all endpoints evaluated in the present study. Here we offer a few potential reasons for this observation:

1. tBHQ is not ideally suited for use as an antioxidant. As noted by Kobayashi et al. [16], tBHQ is metabolized to an electrophilic quinone in cells, suggesting it could cause oxidative stress itself. Furthermore, it has been referenced to as a weak pro-oxidant in previous work studying oxidative stress [30].
2. The concentration of tBHQ (100 µg/L) selected was low enough that no significant differences in mortality or deformities were observed relative to controls, but perhaps not high enough to induce the activation of the antioxidant defense system.
3. The observed results were influenced by the duration and developmental stage of exposure. In the present study, embryos were pre-treated for 4h with 100 µg/L tBHQ immediately following collection. Previous work by Timme-Laragy et al. [17] treated embryos (at 48 or 72 hpf) with tBHQ for 4h. Another study conducted a 6h tBHQ treatment in 7-day-old zebrafish larvae [16], while Arnold et al. [12] pretreated zebrafish embryos (within 2 hpf) for 24h with the antioxidant N-acetylcysteine (NAC). Different exposure regimes will likely result in different responses.

To the best of our knowledge, there is no previous research that has looked at the production of deformities in fish embryos following exposure to these chemicals (tBHQ and tBOOH). Therefore, more research is required to better understand whether these pharmacological agents are helpful/suitable for investigating the mechanism of Se toxicity.

## Figures and Tables

**Table 1:** Total Se concentration in test solutions (mean ±SE, *n* = 9–10) and pooled (35–180 larvae; *n* = 3) *Danio rerio* larvae (5 days post fertilization).

Nominal Concentration (µg/L)	Measured Concentration (µg/L)	Tissue Concentration (µg/g dry mass)
Control	0.351 ± 0.026	2.98 ± 0.073
5	5.13 ± 0.30	34.2 ± 3.5
25	24.8 ± 0.41	144 ± 26
125	124 ± 0.81	264 ± 38
625	615 ± 6.4	291 ± 152 *

* = Due to the high rate of mortality, tissue mass only allowed for an *n* = 2.

## Supplementary Materials

The following are available online at https://www.mdpi.com/2305-6304/7/3/44/s1, Table S1: Target genes and their primer sequences, accession numbers, and efficiencies used for real-time qRT-PCR [10,12,20,21], Figure S1: Mean (±SE) percentage of (A) skeletal deformities (kyphosis and scoliosis) and (B) other deformities (craniofacial, finfold, and edema) in larval zebrafish exposed to increasing concentrations of L-selenomethionine (SeMet) via embryo aqueous exposure. Asterisks represent significant differences compared to the control using a Kruskal–Wallis one-way analysis of variance (ANOVA) by ranks followed by Dunn’s multiple comparisons test (*p* < 0.05); *n* = 9–12 replicates of 20 embryos, Figure S2: Mean (±SE) graduated severity index (GSI) scores of (A) skeletal deformities (kyphosis and scoliosis) and (B) other deformities (craniofacial, finfold, and edema) in larval zebrafish exposed to increasing concentrations of L-selenomethionine (SeMet) via embryo aqueous exposure. Asterisks represent significant differences compared to the control using a Kruskal–Wallis one-way analysis of variance (ANOVA) by ranks followed by a Dunn’s multiple comparisons (*p* < 0.05); *n* = 9–12 replicates of 20 embryos, Figure S3: Mean (±SE) time to hatch in zebrafish exposed to increasing concentrations of L-selenomethionine (SeMet) via embryo aqueous exposure. Asterisks represent significant differences from control using a Kruskal–Wallis one-way analysis of variance (ANOVA) by ranks followed by a Dunn’s multiple comparisons test (*p* < 0.05); *n* = 8–12 replicates of 20 embryos, Figure S4: Mean (±SE) percent hatch of zebrafish exposed to increasing concentrations of L-selenomethionine (SeMet) via embryo aqueous exposure. Asterisks represent significant differences from control using a Kruskal–Wallis one-way analysis of variance (ANOVA) by ranks followed by a Dunn’s multiple comparisons test (*p* < 0.05); *n* = 8–12 replicates of 20 embryos, Figure S5: Mean (±SE) percentage of (A) skeletal deformities (kyphosis and scoliosis) and (B) other deformities (craniofacial, finfold, and edema) in larval zebrafish exposed via embryo aqueous exposure to facility water (control), tBHQ, tBOOH, and two concentrations of SeMet (25 and 125 µg Se/L) either with (tBOOH-t, 25-t, 125-t) or without (tBOOH, 25, 125) a tBHQ pre-treatment. Asterisks represent significant differences from control using a Kruskal–Wallis one-way analysis of variance (ANOVA) by ranks followed by a Dunn’s multiple comparisons test (*p* < 0.05); *n* = 13–33 replicates of 20 embryos, Figure S6: Mean (±SE) graduated severity index (GSI) scores of (A) skeletal deformities (kyphosis and scoliosis) and (B) other deformities (craniofacial, finfold, and edema) in larval zebrafish exposed via embryo aqueous exposure to facility water (control), tBHQ, tBOOH, and two concentrations of SeMet (25 and 125 µg Se/L) either with (tBOOH-t, 25-t, 125-t) or without (tBOOH, 25, 125) a tBHQ pre-treatment. Asterisks represent significant differences from control using a Kruskal–Wallis one-way analysis of variance (ANOVA) by ranks followed by a Dunn’s multiple comparisons test (*p* < 0.05); *n* = 13–33 replicates of 20 embryos, Figure S7: Mean (±SE) time to hatch in zebrafish exposed via embryo aqueous exposure to facility water (control), tBHQ, tBOOH, and two concentrations of SeMet (25 and 125 µg Se/L) either with (tBOOH-t, 25-t, 125-t) or without (tBOOH, 25, 125) a tBHQ pre-treatment. Asterisks represent significant differences compared to the control using a Kruskal–Wallis one-way analysis of variance (ANOVA) by ranks followed by a Dunn’s multiple comparisons test (*p* < 0.05); *n* = 20–33 replicates of 20 embryos, Figure S8: Mean (±SE) percent hatch of zebrafish exposed via embryo aqueous exposure to facility water (control), tBHQ, tBOOH, and two concentrations of SeMet (25 and 125 µg Se/L) either with (tBOOH-t, 25-t, 125-t) or without (tBOOH, 25, 125) a tBHQ pre-treatment. Asterisks represent significant differences compared to the control using a Kruskal–Wallis one-way analysis of variance (ANOVA) by ranks followed by a Dunn’s multiple comparisons test (*p* < 0.05); *n* = 20–33 replicates of 20 embryos, Figure S9: Mean (±SE) fold change in gene expression in zebrafish exposed via embryo aqueous exposure to facility water (control), tBHQ, tBOOH, and two concentrations of SeMet (25 and 125 µg Se/L) either with (tBOOH-t, 25-t, 125-t) or without (tBOOH, 25, 125) a tBHQ pre-treatment. Asterisks represent significant differences compared to the control using either a one-way analysis of variance (ANOVA) followed by Holm-Sidak’s multiple comparisons test or a Kruskal–Wallis one-way ANOVA followed by a Dunn’s multiple comparisons test (*p* < 0.05); *n* = 4–5 replicates of 11–20 zebrafish larvae, Figure S10: Mean (±SE) percentage of lordosis in larval zebrafish exposed to increasing concentrations of L-selenomethionine (SeMet) via embryo aqueous exposure. Asterisks represent significant differences compared to the control using a Kruskal–Wallis one-way analysis of variance (ANOVA) by ranks followed by Dunn’s multiple comparisons test (*p* < 0.05); *n* = 9–12 replicates of 20 embryos, Figure S11: Mean (±SE) percentage of lordosis in larval zebrafish exposed via embryo aqueous exposure to facility water (control), tBHQ, tBOOH, and two concentrations of SeMet (25 and 125 µg Se/L) either with (tBOOH-t, 25-t, 125-t) or without (tBOOH, 25, 125) a tBHQ pre-treatment. Asterisks represent significant differences from control using a Kruskal–Wallis one-way analysis of variance (ANOVA) by ranks followed by a Dunn’s multiple comparisons test (*p* < 0.05); *n* = 13–33 replicates of 20 embryos.
