# Supplementary material for: Toxicity of Aqueous L-Selenomethionine and Tert-Butyl Hydroperoxide Exposure to Zebrafish (Danio rerio) Embryos Following Tert-Butyl Hydroquinone Treatment
Source: Toxics. 2019 Aug 29;7(3):44. doi: 10.3390/toxics7030044 (PMC6789818; doi:10.3390/toxics7030044)
Supplement: Supplementary file 1 [file toxics-07-00044-s001.pdf]

Supplemental Information

Toxicity of Aqueous L-Selenomethionine and Tert-Butyl Hydroperoxide Exposure to Zebrafish (*Danio rerio*) Embryos Following Tert-Butyl Hydroquinone Treatment

Allyson K. Gerhart and David M. Janz

Table S1 Target genes and their primer sequences, accession numbers, and efficiencies used for real time qRT-PCR [10,12,20-21].

Target Gene	Accession #	Primer sequences (5'-3')	Efficiency (%)
nrf2a	NM_182889.1	F: ACACACACCTGAAGCAGACG<br>R: GGCATCATGAGATCAGTGGA	135.054
gpx1a	NM_001007281.2	F: GAAATACGTCCGTCCTGGAA<br>R: CATAAGGGACACAGGGTCGT	106.439
gst p1	NM_131734.3	F: TGGTGCTTTGAAGATCATGC<br>R: CTGAAACAGCACCAGGTCAC	94.656
sod2	NM199976	F: TATGCAGCTTCATCACAGCAAGCA<br>R: GGTTGTCACATCACCCTTGGCC	92.043
gsr	NM001020554	F: ACAGTCAGTGAGGATGATGTGCCAG<br>R: TAGACCCAAGAGTGGAAAGAATACCAGC	101.401
gclc	NM_199277	F: AAGTGGATGAGGGAGTTTGTTGCC<br>R: CTTGTGGAGCAGGTCGTAGTTGAT	96.885
ahr2	AF063446.1	F: CCAGAGCCCTACACAAGCAT<br>R: TCCTTAAGTGGACGGTTTGC	109.186
actb	AF057040	F: AAG ATC AAG ATC ATT GCT CCC<br>R: CCA GAC TCA TCG TAC TCC T	101.269
EF1a	NM_131263.1	F: CTTCAACGCTCAGGTCATCA<br>R: CGGTCGATCTTCTCCTTGAG	98.262

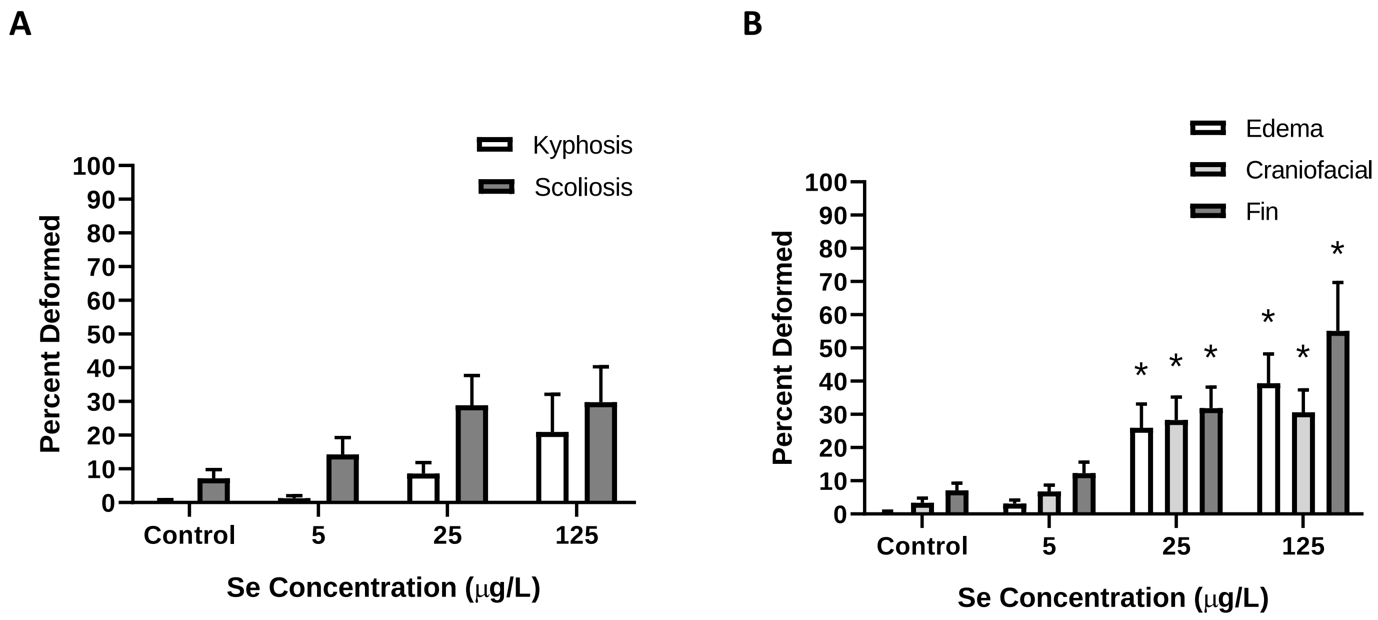

Figure S1. Mean (±SE) percentage of (A) skeletal deformities (kyphosis and scoliosis) and (B) other deformities (craniofacial, finfold, and edema) in larval zebrafish exposed to increasing concentrations of L-selenomethionine (SeMet) via embryo aqueous exposure. Asterisks represent significant differences compared to the control using a Kruskal–Wallis one-way analysis of variance (ANOVA) by ranks followed by Dunn’s multiple comparisons test ($p < 0.05$); n = 9-12 replicates of 20 embryos.

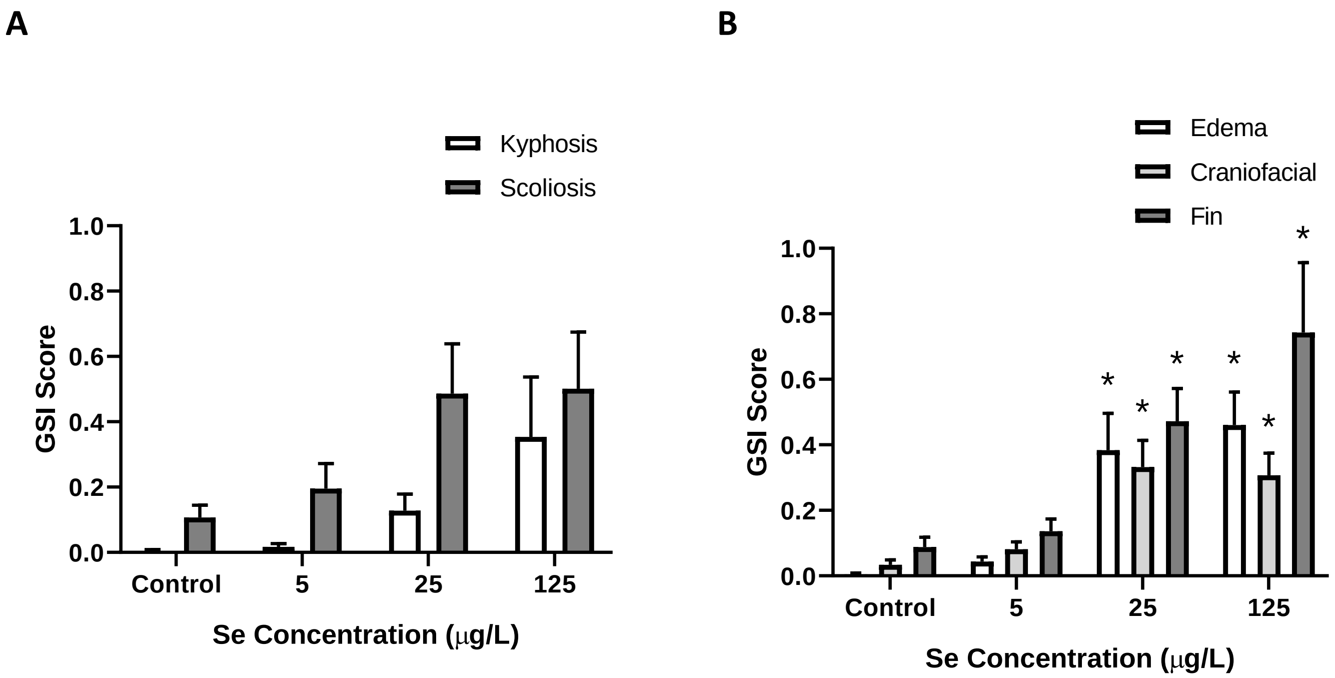

Figure S2. Mean (±SE) graduated severity index (GSI) scores of (A) skeletal deformities (kyphosis and scoliosis) and (B) other deformities (craniofacial, finfold, and edema) in larval zebrafish exposed to increasing concentrations of L-selenomethionine (SeMet) via embryo aqueous exposure. Asterisks represent significant differences compared to the control using a Kruskal–Wallis one-way analysis of variance (ANOVA) by ranks followed by a Dunn's multiple comparisons ($p < 0.05$); n = 9-12 replicates of 20 embryos.

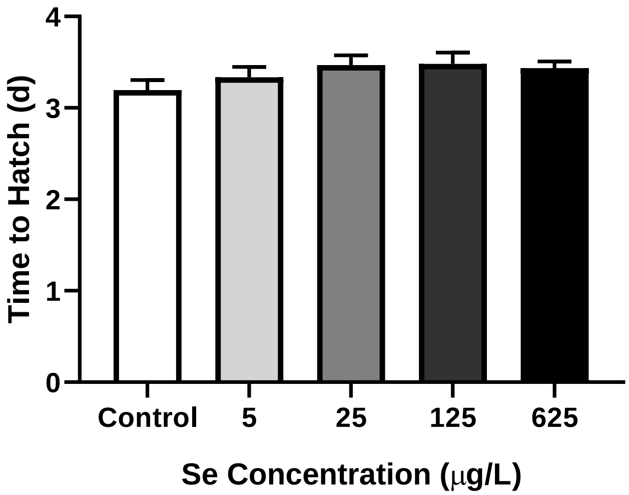

Figure S3. Mean (± SE) time to hatch in zebrafish exposed to increasing concentrations of L-selenomethionine (SeMet) via embryo aqueous exposure. Asterisks represent significant differences from control using a Kruskal–Wallis one-way analysis of variance (ANOVA) by ranks followed by a Dunn's multiple comparisons test ($p < 0.05$); n = 8-12 replicates of 20 embryos.

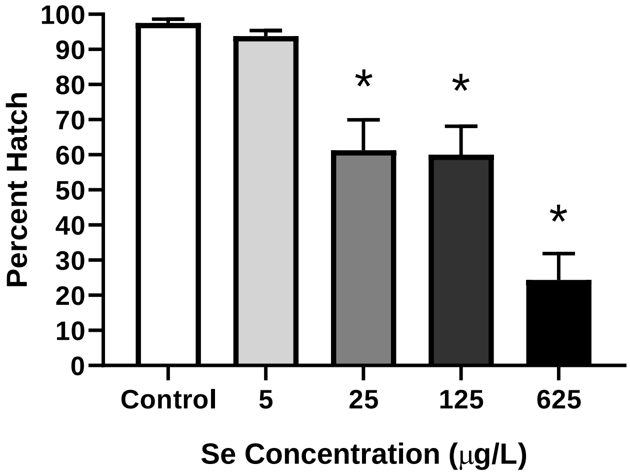

Figure S4. Mean (± SE) percent hatch of zebrafish exposed to increasing concentrations of L-selenomethionine (SeMet) via embryo aqueous exposure. Asterisks represent significant

differences from control using a Kruskal–Wallis one-way analysis of variance (ANOVA) by ranks followed by a Dunn's multiple comparisons test ($p < 0.05$); n = 8-12 replicates of 20 embryos.

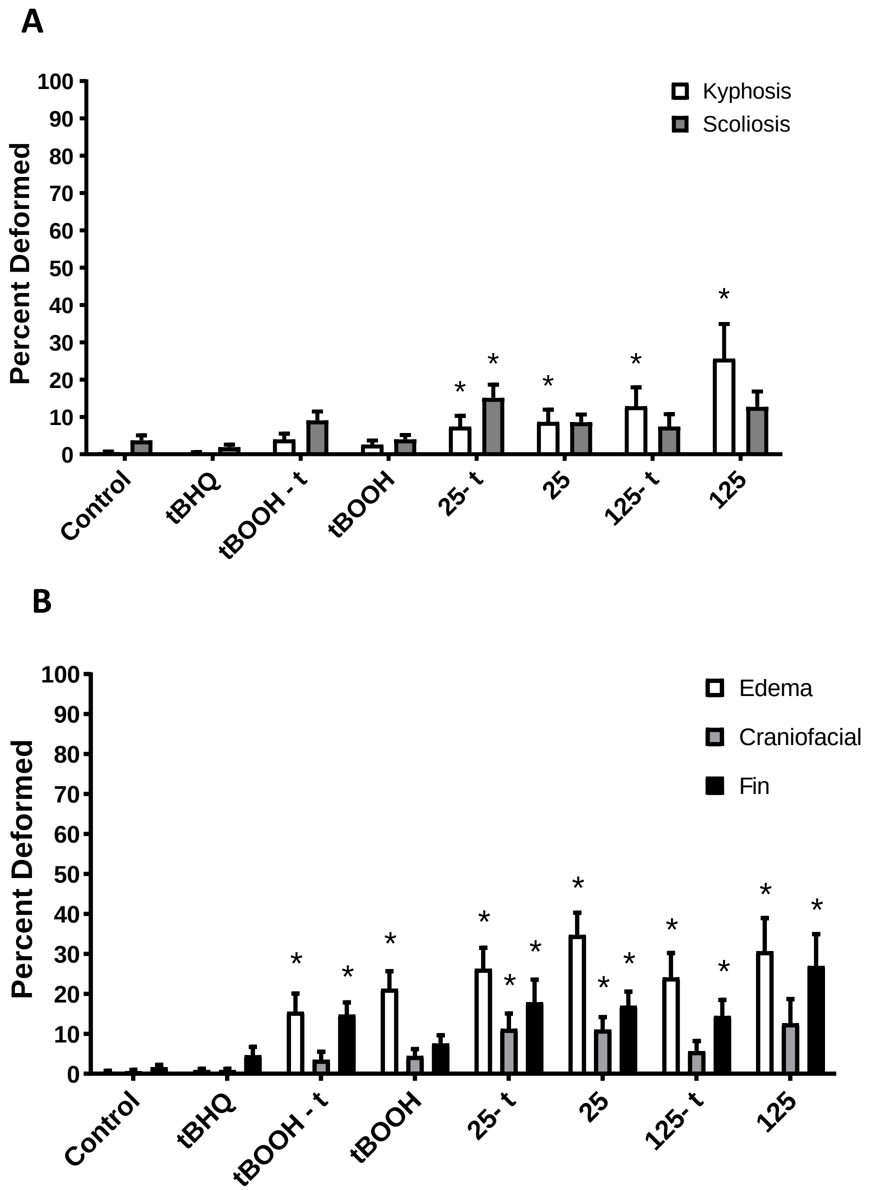

Figure S5. Mean (±SE) percentage of (A) skeletal deformities (kyphosis and scoliosis) and (B) other deformities (craniofacial, finfold, and edema) in larval zebrafish exposed via embryo

aqueous exposure to facility water (control), tBHQ, tBOOH, and two concentrations of SeMet (25 and 125 µg Se/L) either with (tBOOH-t, 25-t, 125-t) or without (tBOOH, 25, 125) a tBHQ pre-treatment. Asterisks represent significant differences from control using a Kruskal–Wallis one-way analysis of variance (ANOVA) by ranks followed by a Dunn's multiple comparisons test ($p < 0.05$); n = 13-33 replicates of 20 embryos.

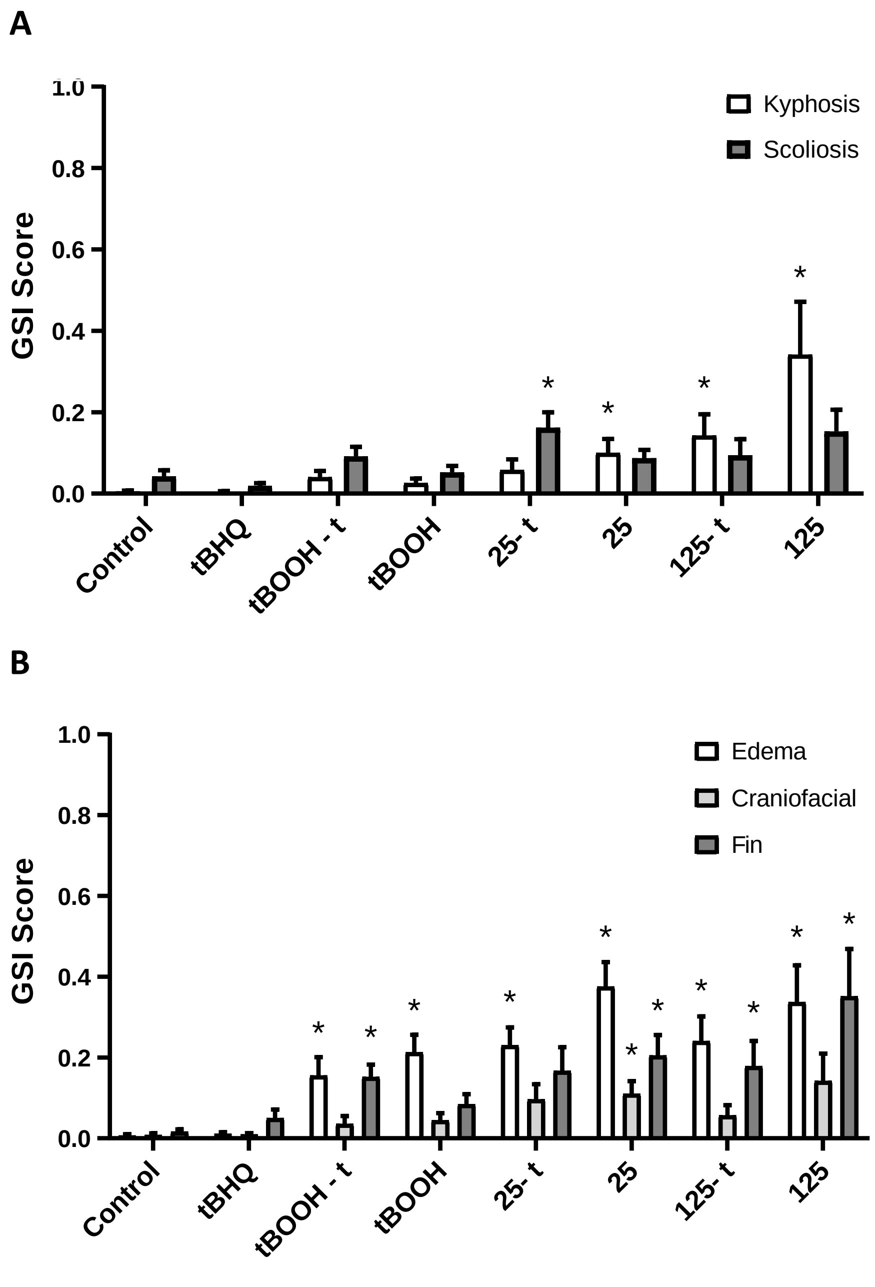

Figure S6. Mean (±SE) graduated severity index (GSI) scores of (A) skeletal deformities (kyphosis and scoliosis) and (B) other deformities (craniofacial, finfold, and edema) in larval zebrafish exposed via embryo aqueous exposure to facility water (control), tBHQ, tBOOH, and two concentrations of SeMet (25 and 125 µg Se/L) either with (tBOOH-t, 25-t, 125-t) or without (tBOOH, 25, 125) a tBHQ pre-treatment. Asterisks represent significant differences from control using a Kruskal–Wallis one-way analysis of variance (ANOVA) by ranks followed by a Dunn's multiple comparisons test ($p < 0.05$); n = 13-33 replicates of 20 embryos.

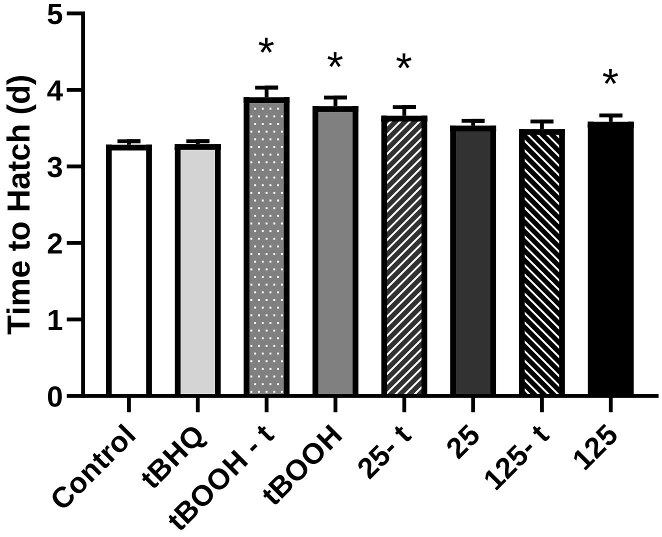

Figure S7. Mean (± SE) time to hatch in zebrafish exposed via embryo aqueous exposure to facility water (control), tBHQ, tBOOH, and two concentrations of SeMet (25 and 125 µg Se/L) either with (tBOOH-t, 25-t, 125-t) or without (tBOOH, 25, 125) a tBHQ pre-treatment. Asterisks represent significant differences compared to the control using a Kruskal–Wallis one-way analysis of variance (ANOVA) by ranks followed by a Dunn's multiple comparisons test ($p < 0.05$); n = 20-33 replicates of 20 embryos.

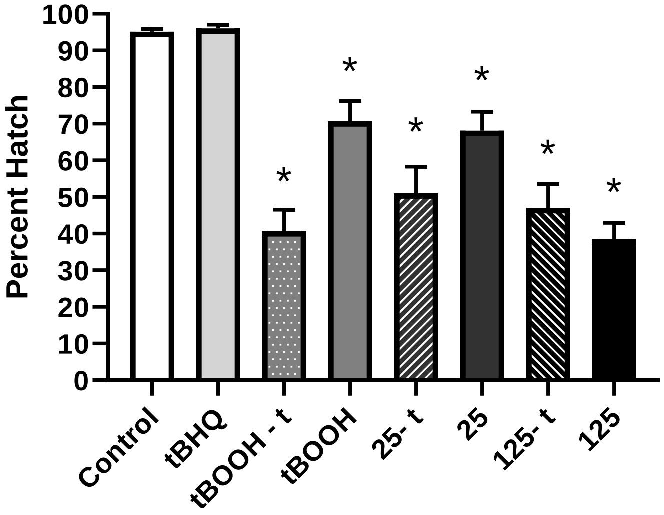

Figure S8. Mean (± SE) percent hatch of zebrafish exposed via embryo aqueous exposure to facility water (control), tBHQ, tBOOH, and two concentrations of SeMet (25 and 125 µg Se/L) either with (tBOOH-t, 25-t, 125-t) or without (tBOOH, 25, 125) a tBHQ pre-treatment. Asterisks represent significant differences compared to the control using a Kruskal–Wallis one-way analysis of variance (ANOVA) by ranks followed by a Dunn's multiple comparisons test ($p < 0.05$); n = 20-33 replicates of 20 embryos.

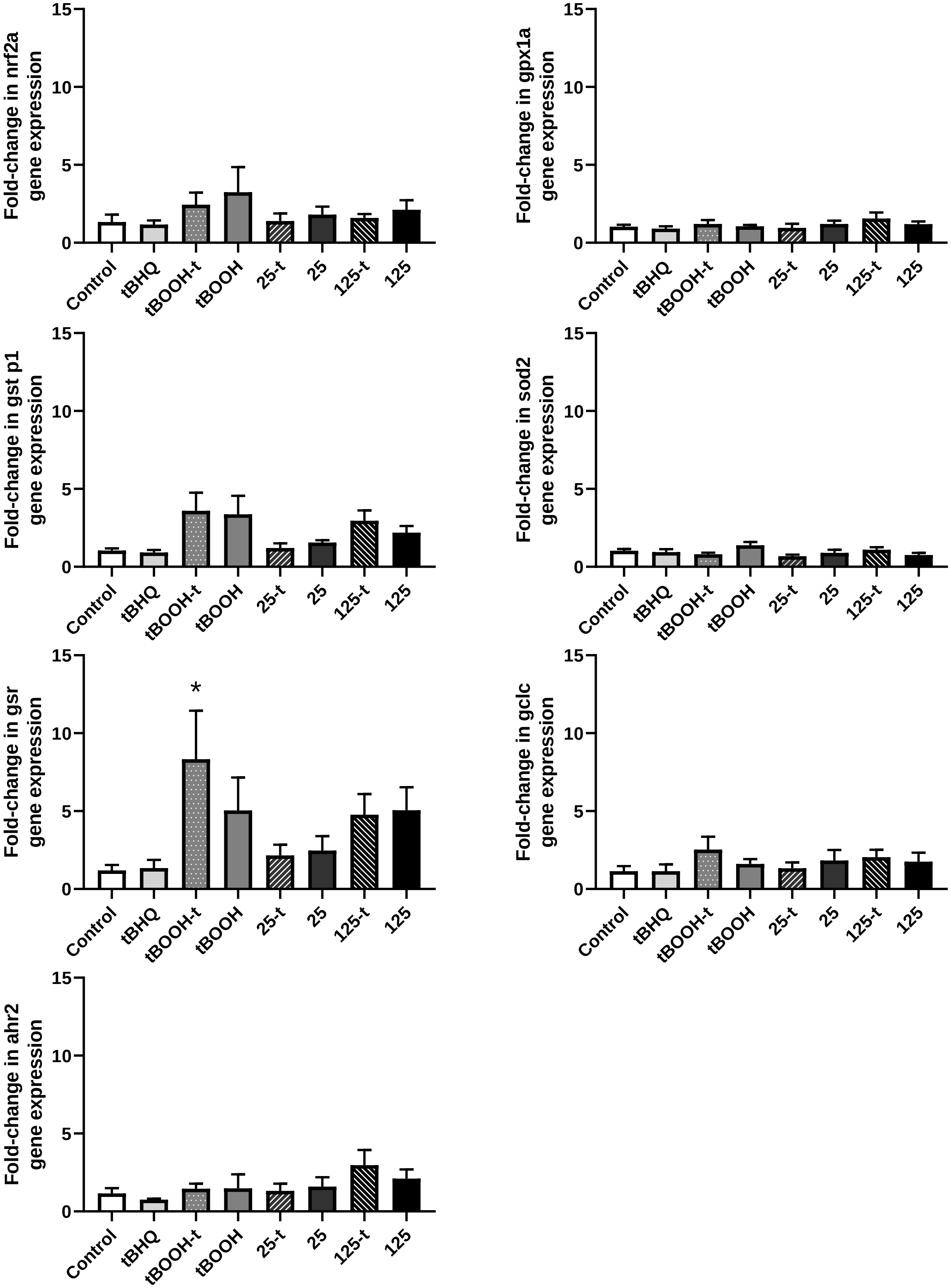
Fold-change in nrf2a gene expression
Fold-change in gpx1a gene expression
Fold-change in gst p1 gene expression
Fold-change in sod2 gene expression
Fold-change in gsr gene expression
Fold-change in gclc gene expression
Fold-change in ahr2 gene expression
0
5
10
15
*
Control
tBHQ
tBOOH-t
tBOOH
25-t
25
125-t
125

Figure S9. Mean (± SE) fold change in gene expression in zebrafish exposed via embryo aqueous exposure to facility water (control), tBHQ, tBOOH, and two concentrations of SeMet (25 and 125 µg Se/L) either with (tBOOH-t, 25-t, 125-t) or without (tBOOH, 25, 125) a tBHQ pre-treatment. Asterisks represent significant differences compared to the control using either a one-way analysis of variance (ANOVA) followed by Holm-Sidak's multiple comparisons test or a Kruskal–Wallis one-way ANOVA followed by a Dunn's multiple comparisons test ($p < 0.05$); n = 4-5 replicates of 11-20 zebrafish larvae.

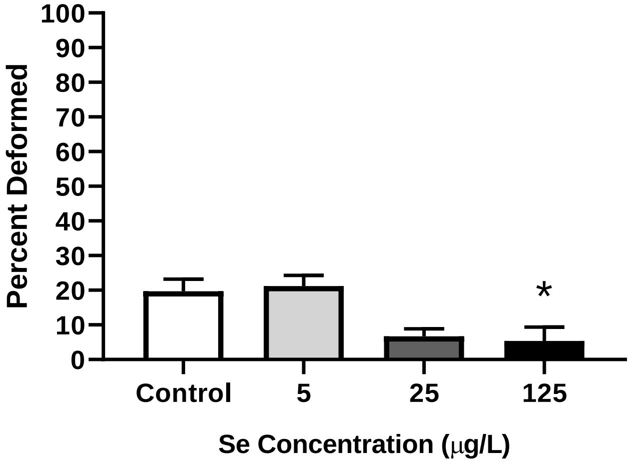

Figure S10. Mean (±SE) percentage of lordosis in larval zebrafish exposed to increasing concentrations of L-selenomethionine (SeMet) via embryo aqueous exposure. Asterisks represent significant differences compared to the control using a Kruskal–Wallis one-way analysis of variance (ANOVA) by ranks followed by Dunn's multiple comparisons test ($p < 0.05$); n = 9-12 replicates of 20 embryos.

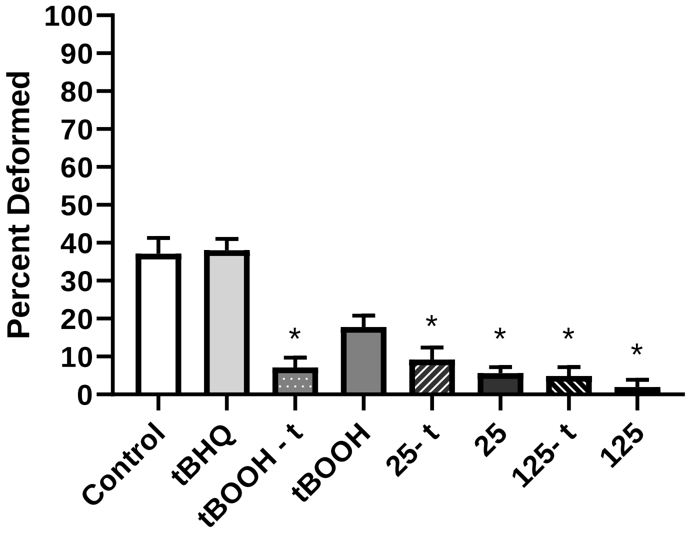

Figure S11. Mean (±SE) percentage of lordosis in larval zebrafish exposed via embryo aqueous exposure to facility water (control), tBHQ, tBOOH, and two concentrations of SeMet (25 and 125 µg Se/L) either with (tBOOH-t, 25-t, 125-t) or without (tBOOH, 25, 125) a tBHQ pre-treatment. Asterisks represent significant differences from control using a Kruskal–Wallis one-way analysis of variance (ANOVA) by ranks followed by a Dunn's multiple comparisons test ($p < 0.05$); n = 13-33 replicates of 20 embryos.
